# Pharmacological modulation of prostaglandin E_2_ (PGE_2_) EP receptors improves cardiomyocyte function under hyperglycemic conditions

**DOI:** 10.14814/phy2.15212

**Published:** 2022-04-10

**Authors:** Karin J. Bosma, Monica Ghosh, Spencer R. Andrei, Lin Zhong, Jennifer C. Dunn, Valerie F. Ricciardi, Juliann B. Burkett, Antonis K. Hatzopoulos, Derek S. Damron, Maureen Gannon

**Affiliations:** ^1^ Department of Veterans Affairs Tennessee Valley Authority Nashville Tennessee USA; ^2^ Department of Medicine Vanderbilt University Medical Center Nashville Tennessee USA; ^3^ Department of Biological Sciences School of Biomedical Sciences Kent State University Kent Ohio USA; ^4^ Department of Molecular Physiology and Biophysics Vanderbilt University Nashville Tennessee USA; ^5^ Department of Cell and Developmental Biology Vanderbilt University Nashville Tennessee USA

**Keywords:** contractility, diabetic cardiomyopathy, ejection fraction, prostaglandin receptor, Type 2 diabetes

## Abstract

Type 2 diabetes (T2D) affects >30 million Americans and nearly 70% of individuals with T2D will die from cardiovascular disease (CVD). Circulating levels of the inflammatory signaling lipid, prostaglandin E_2_ (PGE_2_), are elevated in the setting of obesity and T2D and are associated with decreased cardiac function. The EP3 and EP4 PGE_2_ receptors have opposing actions in several tissues, including the heart: overexpression of EP3 in cardiomyocytes impairs function, while EP4 overexpression improves function. Here we performed complementary studies in vitro with isolated cardiomyocytes and in vivo using db/db mice, a model of T2D, to analyze the effects of EP3 inhibition or EP4 activation on cardiac function. Using echocardiography, we found that 2 weeks of systemic treatment of db/db mice with 20 mg/kg of EP3 antagonist, beginning at 6 weeks of age, improves ejection fraction and fractional shortening (with no effect on heart rate). We further show that either EP3 blockade or EP4 activation enhances contractility and calcium cycling in isolated mouse cardiomyocytes cultured in both normal and high glucose. Thus, peak [Ca^2+^]_I_ transient amplitude was increased, while time to peak [Ca^2+^]_I_ and [Ca^2+^]_I_ decay were decreased. These data suggest that modulation of EP3 and EP4 activity has beneficial effects on cardiomyocyte contractility and overall heart function.

## Graphical abstract

Circulating levels of PGE2 are elevated in obesity and T2D and are associated with decreased cardiac function. The EP3 and EP4 PGE2 receptors have opposing actions in the heart: overexpression of EP3 in cardiomyocytes impairs function, while EP4 overexpression improves function. We show that treatment of T2D mice or cardiomyocytes cultured in high glucose with a PGE2 receptor modulators improves cardiac contractility and calcium cycling in mouse cardiomyocytes cultured in high glucose.

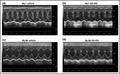

## 1 INTRODUCTION

Type 2 diabetes (T2D) is caused by insufficient functional β‐cell mass in the face of insulin resistance and currently affects more than 30 million Americans. T2D strongly predisposes individuals toward cardiovascular disease (CVD), even when normalized for BMI, age, and other risk factors such as obesity (Abbasi, Brown, Lamendola, McLaughlin & Reaven, 2002). This increased risk for CVD results in nearly 70% of individuals with T2D dying from heart disease or stroke, and is present even in the absence of other risk factors of cardiac dysfunction such as hypertension (Simone et al., 2010). The ventricular dysfunction present in individuals with diabetes in the absence of hypertension or coronary artery disease is termed diabetic cardiomyopathy (DCM) (Bugger & Abel, 2014). DCM is associated with increased left ventricular mass and wall thickness accompanied by initial diastolic and subsequent systolic dysfunction leading to heart failure with preserved ejection fraction (Devereux et al., 2000). Multiple molecular mechanisms have been proposed to contribute to the dysfunction in human DCM, and there remains a critical need for therapeutic targets that can improve cardiac function in the setting of T2D.

The db/db mouse model, long used to study mechanisms of disease in T2D, has also been verified as a model of DCM (Alex, Russo, Holoborodko & Frangogiannis, 2018). Although there have been conflicting descriptions of the cardiac phenotype in db/db mice, depending on the specific genetic background and age of animals used, in general, db/db mice have evidence of cardiomyocyte hypertrophy, fibrosis, and diastolic dysfunction (Alex et al., 2018). In db/db mice on a C57BLKS/J background, systolic volume increases at around 8 weeks of age, with dilation occurring by 16 weeks (Baumgardt et al., 2016; Nielsen et al., 2009). Reduced fractional shortening and ejection fraction were also noted in this model between 12 and 16 weeks of age (Baumgardt et al., 2016; Plante et al., 2014, 2015). This phenotype mimics that of human DCM, where structural changes in the heart, including hypertrophy and fibrosis, are often followed by diastolic dysfunction, and eventually systolic dysfunction (Jia, Hill & Sowers, 2018). Additionally, studies on isolated cardiomyocytes from db/db mice found a decreased peak shortening amplitude, decreased cell shortening and relengthening amplitudes, and an increased Ca^2+^ decay rate (Kralik, Ye, Metreveli, Shen & Epstein, 2005). Another study found resting diastolic Ca^2+^ levels were reduced in db/db mice, transient Ca^2+^ decay was increased, and the height of the Ca^2+^ transient was reduced (Belke, Swanson & Dillmann, 2004).

T2D is associated with low‐grade inflammation, which occurs concomitantly with elevated prostaglandin (PG) synthesis (Sjoholm & Nystrom, 2006), including prostaglandin E_2_ (PGE_2_). PGE_2_ exerts its mechanisms of action through four G protein‐coupled receptors (GPCRs) termed E‐Prostanoid (EP1‐4) receptors (Hata, & Breyer, 2004; Vennemann et al., 2012). Signaling via Gi‐coupled EP3 inhibits adenylate cyclase and decreases cAMP levels whereas Gs‐coupled EP4 results in activated adenylate cyclase and increased cAMP. Thus, EP3 and EP4 often have opposite effects in the same cell type. For example, our group previously showed that EP3 and EP4 play opposing roles in pancreatic insulin‐producing β‐cell proliferation and survival in islets from mice and humans (Carboneau et al., 2017). Similarly, studies in the heart reveal that EP3 and EP4 have opposite effects on cardiac function (Bryson et al., 2018; Gu et al., 2016; Hishikari et al., 2009; Meyer‐Kirchrath et al., 2009).

Given the correlation of the canonical cAMP/protein kinase A (PKA) pathway in cardiac tissue with regulating cardiac function (Guellich, Mehel & Fischmeister, 2014), several studies have explored the extent to which EP3 or EP4 regulates and/or modulates cardiac contractile parameters. PGE_2_ decreases cardiomyocyte contractility and cardiac function via EP3 activation (Gu et al., 2016). Transgenic overexpression of EP3 in cardiomyocytes using the myosin heavy chain promoter results in severely diminished ejection fraction concomitant with elevated end‐diastolic and end‐systolic ventricular volume following ischemic injury (Meyer‐Kirchrath et al., 2009). In contrast, EP4 overexpression improves fractional shortening, ejection fraction, and left ventricular internal diameter during systole (Bryson et al., 2018). Additionally, pharmacological activation of EP4 results in improved cardiomyocyte contractile parameters and improved cardiac function following ischemic injury (Gu et al., 2016), whereas mice with cardiac‐specific EP4 inactivation exhibit reduced cardiac function following myocardial ischemia (Qian et al., 2008).

Although strides have been made with regards to understanding the roles of EP3 and EP4 in the regulation of cardiac contractility, the extent to which EP3 or EP4 modulation alters cardiomyocyte contractility or cardiac function in the setting of hyperglycemia or T2D remains unexplored. In the current study we sought to address if EP3 blockade or EP4 activation could improve cardiomyocyte function under hyperglycemic conditions, and if treatment with an EP3 antagonist in vivo could improve cardiac function in the db/db mouse model of T2D. We found that inhibition of EP3 or activation of EP4 in cultured isolated mouse cardiomyocytes improved contractile parameters and Ca^2+^ handling. Additionally, we found that cardiac function was impaired in 8‐week‐old db/db mice, and that 2 weeks of treatment with an EP3 antagonist in vivo caused modest improvements in cardiac function.

## 2 MATERIALS AND METHODS

### 2.1 Animal models

C57BL6/J wild‐type, db/+, or db/db male mice (Jackson Laboratories) were utilized and maintained in accordance with the *Guide for the Care and Use of Laboratory Animals* (NIH). Since males develop more predictable and severe diabetes in this model, 6‐week‐old male mice were injected daily for 2 weeks with 20 mg/kg DG‐041 or volume‐matched vehicle (phosphate‐buffered saline [PBS] +10% dimethyl sulfoxide [DMSO]) subcutaneously. Mice were housed in the Kent State (Kent, OH) or Vanderbilt University Medical Center (Nashville, TN) animal care facility, both of which are accredited by the American Association for Accreditation of Laboratory Animal Care. All mouse experiments were approved by the Institutional Animal Care and Use Committee of Kent State or Vanderbilt University Medical Center.

### 2.2 Cardiomyocyte isolation

Hearts were excised from 4‐month‐old wild‐type or db/db mice, prepared for aortic cannulation and transferred to a Langendorff apparatus for cardiomyocyte isolation, as previously described (Andrei, Ghosh, Sinharoy & Damron, 2019; Andrei et al., 2017). In brief, hearts underwent retrograde perfusion at 37°C with a modified Krebs‐Henseleit buffer (in mM: 120.4 NaCl, 4.8 KCl, 0.6 KH_2_PO_4_, 0.6 Na_2_HPO_4_, 1.2 MgSO_4_‐7H_s_O, 10 Na‐HEPES, 4.6 NaHCO_3_, 30 taurine, 10 BDM, and 5.5 glucose, pH 7.4). The Krebs‐Henseleit buffer was sterile‐filtered and paced at a rate of 4 ml/min. After perfusion for 4 min, the digestion buffer containing collagenase type II (300 U/mg, Worthington Biochemical) perfused the heart for an additional 5–7 min until the heart became spongy. The left ventricles were removed, minced, and triturated in Krebs‐Henseleit buffer containing fetal bovine serum. The resulting cellular digest was washed and resuspended at 23°C in HEPES‐buffered saline (in mM: 118 NaCl, 4.8 KCl, 0.6 KH_2_PO_4_, 4.6 NaHCO_3_, 0.6 NaH_2_PO_4_, 5.5 glucose, pH 7.4). Cardiomyocyte yield was consistently ~90%.

### 2.3 Simultaneous measurement of intracellular free Ca^2+^ concentration ([Ca^2+^]_i_) and sarcomere shortening

Simultaneous measurement of intracellular Ca^2+^ concentration [Ca2+]_i_ and contractile function was performed in individual freshly isolated cardiomyocytes as previously described (Andrei et al., 2017, 2019). Cardiomyocytes were cultured in normal glucose (NG) conditions (5.6 mM) or high glucose (HG) conditions (16.7 mM) and incubated at room temperature for 2 h prior to loading fura‐2 acetoxy methylester (fura‐2/AM; 2 µM) in HEPES‐buffered saline (in mM: 118 NaCl, 4.8 KCl, 1.23 CaCl_2_, 0.8 MgSO_4_‐7H2O, 0.6 KH_2_PO_4_, 4.6 NaHCO_3_, 0.6 NaH_2_PO_4_, 5.5 glucose, pH 7.4) for 25 min at room temperature. Coverslips containing the fura‐2‐loaded cardiomyocytes were then mounted on the stage of an Olympus IX‐71 inverted fluorescence microscope (Olympus America), superfused continuously with HEPES‐buffered saline at a flow rate of 2 mL/min, and paced at a frequency of 0.3 Hz (30 V, 5 ms duration). Following baseline contractility calibration, cardiomyocytes were treated with the EP3 antagonist, DG‐041 (30 nM) or the EP4 agonist, CAY10598 (100 nM) for ~5 min or when the contractility reached a clear “plateau” effect. Sarcomere shortening and [Ca^2+^]_i_ measurements were simultaneously recorded on individual cells using the fluorescence imaging system and Easy Ratio Pro software (Photon Technology International) equipped with a multiwavelength spectrofluorometer (Deltascan RFK6002) and a QuantEM 512SC electron multiplying camera (Photometrics). Images and real‐time Ca^2+^ tracing data were acquired using an alternating excitation wavelength protocol (340, 380 nm/20 Hz) and emission wavelength of 510 nm. Background fluorescence was automatically corrected for the experiments using Easy Ratio Pro. The ratio of the two intensities were used to measure changes in [Ca^2+^]_i_. Hardware and software for data acquisition and analysis were generously provided by Horiba Scientific.

### 2.4 Analysis of [Ca^2+^]_i_ and shortening data

The following variables were calculated for each individual cardiomyocyte contraction: sarcomere length (µm), fractional shortening (% of sarcomere length change during shortening), maximum velocity of cell shortening and relengthening (µm/sec), peak [Ca^2+^]_i_ (340/380 ratio), and [Ca^2+^]_i_ decay to baseline (ms). Variables from 10 contractions were averaged to obtain mean values at baseline and in response to the intervention since averaging the variables over time minimizes beat‐to‐beat variation. The summarized shortening raw data are expressed as % change in sarcomere length (fractional shortening) and mm/sec (velocity of shortening/relengthening). The summarized [Ca^2+^]_i_ raw data are expressed in msec. Individual [Ca^2+^]_i_ transient traces were smoothed using the Savitzky‐Golay filter to increase the signal‐to‐noise ratio and enhance the clarity of the figure to highlight changes in timing parameters.

### 2.5 Transthoracic echocardiography

In vivo cardiac functional parameters were evaluated with transthoracic echocardiography in the conscious state using a Vevo2100 Imaging System (VisualSonics Inc) before and 14 days following the respective treatment protocol. Pre‐warmed echo transmission gel was applied to the shaved chest wall prior to the acquisition of echo images, and parasternal long‐ and short‐axis view at the papillary muscle level and 2‐D guided M‐mode images were recorded. Left ventricular dimension in systole (LVIDs) and diastole (LVIDd), systolic and diastolic interventricular septum thickness (IVSs, IVSd), systolic and diastolic posterior wall thickness (LVPWs, LVPWd), ejection fraction, and fractional shortening were measured in three consecutive beats according to the guidelines and standards of American Society of Echocardiography leading edge method (Lang et al., 2005). Qualitative and quantitative measurements were calculated by blinded reviewers using the VisualSonics VEVO 2100 Imaging System software.

### 2.6 Analysis of gene expression

RNA was isolated from whole mouse hearts using the Qiagen RNeasy kit. cDNA was generated using the High Capacity cDNA Reverse Transcription kit (Applied Biosciences). Gene expression was then quantitated by PCR using the iQ SYBR Green Supermix (Bio‐Rad). Fold induction was calculated using the 2^−ΔΔCt^ method, and the expression of genes of interest were normalized to actin. The primers used have been previously described (Carboneau et al., 2017).

### 2.7 Statistical analysis

All experimental protocols were repeated in a minimum of three different mice. Exact numbers used are indicated in figure legends. Within group comparisons were made using two‐way ANOVA and Tukey multiple comparisons post hoc test. Differences were considered statistically significant at *p* < 0.05. All results are expressed as mean +/‐ SEM. Statistical analysis was conducted using GraphPad Prism.

## 3 RESULTS

### 3.1 Contractility and Ca^2+^ cycling dynamics are impaired in cardiomyocytes isolated from db/db mice

**FIGURE 1:**
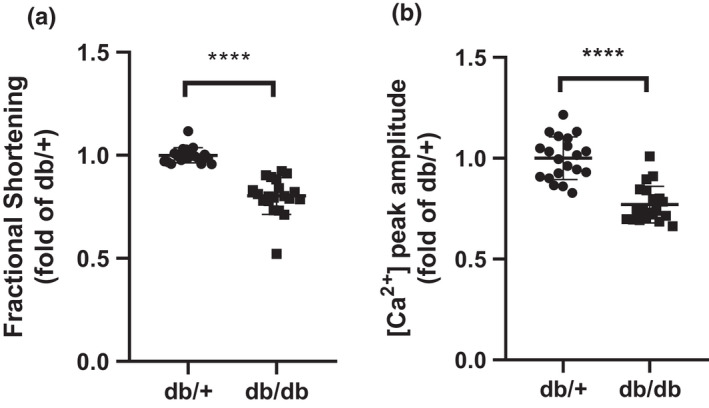
Contractility and Ca^2+^ peak amplitude are diminished in adult ventricular cardiomyocytes from db/db mice. (a) Fractional shortening (µm/sec) and (b) intracellular Ca^2+^ concentration ([Ca^2+^]_i_) peak amplitude (340/380 ratio) are significantly attenuated in cardiomyocytes isolated from 4‐month‐old db/db mice compared to db/+ mice. ●, db/+. ◾, db/db. ****p < 0.001 versus db/+. Data were analyzed using Student's *t*‐test and Bonferroni post hoc analysis. Results are expressed as a fold of the db/+ control. *N* = 20 cardiomyocytes from five hearts per group

Mice homozygous for a spontaneous mutation in the leptin receptor (db/db) become obese and hyperglycemic at an early age (4–6 weeks), and are frequently used as a model to study T2D mechanisms and its associated complications. As in humans with obesity and T2D, db/db mice have elevated levels of PGE_2_ (Sun et al., 2013). To determine the potential effects of the diabetic environment on cardiomyocyte function, cardiomyocytes isolated from hearts from 4‐month‐old db/+ or db/db mice were electrically paced using a voltage stimulator and assessed for alterations in contractile function and Ca^2+^ cycling dynamics. Fractional shortening and [Ca^2+^]_i_ peak amplitudes were used as the primary indicators for contractile function and Ca^2+^ cycling dynamics, respectively. Cardiomyocytes from db/db mice displayed worsened fractional shortening (1.00 +/− 0.01 in db/+ vs. 0.80 +/− 0.02 in db/db; *p *< 0.0001) and diminished [Ca^2+^]_i_ peak amplitudes (1.00 +/− 0.02 in db/+ vs. 0.77 +/− 0.02 in db/db; *p *< 0.0001) when compared to their db/+ counterparts (Figure 1). These results support previously published studies describing impaired cardiomyocyte function in mice with T2D or streptozotocin‐induced diabetes, including decreased contractility and Ca^2+^ cycling dynamics (Belke et al., 2004; Kralik et al., 2005; Wickley, Shiga, Murray & Damron, 2007).

### 3.2 EP3 blockade improves cardiomyocyte contractile function and Ca^2+^ cycling under normal and high glucose conditions

**FIGURE 2:**
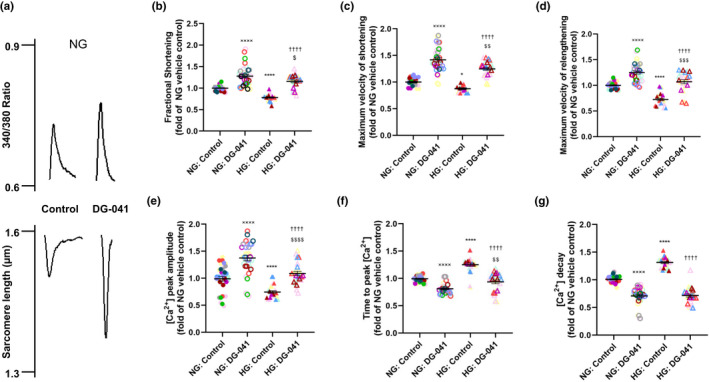
EP3 blockade improves contractility and Ca^2+^ cycling in cardiomyocytes. Isolated cardiomyocytes from 4‐month‐old wild‐type mice were cultured in normal glucose (NG) and high glucose (HG). (a) Representative traces of calcium flux (upper panel) and sarcomere length (lower panel) of cardiomyocytes cultured in low glucose, with or without the EP3 antagonist DG‐041. Acute hyperglycemia resulted in significant decreases in (b) fractional shortening, (c) maximal velocity of shortening, (d) maximal velocity of relengthening, and (e) Ca^2+^ peak amplitude. Additionally, acute hyperglycemia resulted in significant increases in (f) time to peak [Ca^2+^]_I_ and (G) Ca^2+^]_I_ decay. Acute treatment with the EP3 antagonist DG‐041 resulted in increased (b) fractional shortening (percent of relaxed sarcomere length), (c) maximum velocity of shortening (‐µm/sec), and (d) maximum velocity of relengthening (+µm/s). EP3 blockade also increased (e) [Ca^2+^]_i_ peak amplitude (340/380 ratio) and accelerated (f) time to peak [Ca^2+^]_i_ (s), and (G) [Ca^2+^]_i_ decay (s). Results are expressed as a fold of the steady‐state baseline for the vehicle‐treated normal glucose control (NG: Control). Same colored symbols represent separate individual cardiomyocytes derived from the same mouse heart. *N* = 20 cardiomyocytes from five hearts per group. Data were analyzed using a two‐way ANOVA with Tukey multiple comparisons post hoc test. ●: NG control, ⚪: NG DG‐041, ▴: HG control, ▵: HG DG‐041. x = vs NG control, * vs NG control, ^†^ vs HG control, ^$^ vs NG DG041. 1 symbol: *p *< 0.05, 2 symbols: *p *< 0.01, 3 symbols: *p *< 0.001, 4 symbols: *p *< 0.0001

Previous work has shown that hyperglycemia can interfere with the formation and maintenance of contractile structures in cultured ventricular adult rat cardiomyocytes (Dyntar et al., 2006). To determine the effects of acute hyperglycemia on cardiomyocyte function, cardiomyocytes were isolated from 4‐month‐old wild‐type mice, cultured in normal glucose (5.6 mM) or hyperglycemic (16.7 mM) conditions, and electrically paced with a field stimulator. Representative traces are shown in Figure 2a. Acute hyperglycemia resulted in significant decreases in fractional shortening (Figure 2b), maximal velocity of shortening (Figure 2c), maximal velocity of relengthening (Figure 2d), and Ca^2+^ peak amplitude (Figure 2e). Additionally, acute hyperglycemia resulted in significant increases in time to peak [Ca^2+^]_I_ (Figure 2f) and [Ca^2+^]_I_ decay (Figure 2g). Importantly, culturing wild‐type cardiomyocytes under hyperglycemic conditions induced alterations in contractile parameters and Ca^2+^ cycling dynamics that resemble the disparities observed between db/+ and db/db animals in vivo (decreased fractional shortening and decreased Ca^2+^ peak amplitude).

Since chronically decreased EP3 activity improves measures of cardiac function in vivo (Meyer‐Kirchrath et al., 2009), we investigated whether acute EP3 receptor blockade alters contractility or Ca^2+^ cycling in isolated cardiomyocyte under normo‐ and hyperglycemic conditions. Cardiomyocytes isolated from 4‐month‐old wild‐type mice were cultured in normal glucose (5.6 mM) or hyperglycemic (16.7 mM) conditions and electrically paced with a field stimulator before being superfused with the EP3 antagonist, DG‐041, or vehicle; representative traces are shown in Figure 2a. In DG‐041‐treated cardiomyocytes cultured in high glucose, fractional shortening was increased (1.16 +/− 0.03 vs 0.78 +/− 0.02; Figure 2b), and both maximum velocity of shortening (1.25 +/− 0.03 vs 0.88 +/− 0.01; Figure 2c) and maximum velocity of relengthening were accelerated (1.07 +/− 0.04 vs 0.72 +/− 0.03; Figure 2d) when compared to the vehicle‐treated controls. Similar results were found in cardiomyocytes cultured in normal glucose (Figure 2b–d). Additionally, peak [Ca^2+^]_I_ transient amplitude was increased (1.09 +/− 0.04 vs 0.74 +/− 0.02; Figure 2e) while time to peak [Ca^2+^]_I_ (0.94 +/− 0.03 vs 1.25 +/− 0.03; Figure 2f) and [Ca^2+^]_I_ decay (0.72 +/− 0.03 vs 1.31 +/− 0.02; Figure 2g) were decreased in DG‐041‐treated cardiomyocytes cultured in high glucose; similar results were observed in normal glucose‐cultured cells (Figure 2e–g). Taken together, these data suggest that the in vitro model of acute high glucose culture used in this study mimics the effects of a T2D environment on cardiomyocyte function.

### 3.3 Ca^2+^ dynamics and contractile function are increased following EP4 activation in normal and high glucose conditions

**FIGURE 3:**
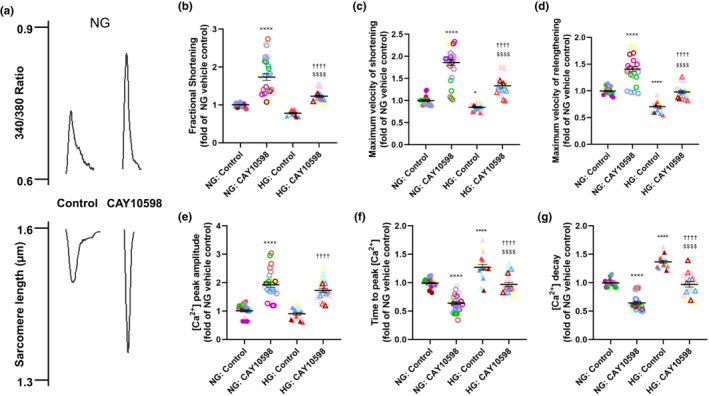
EP4 activation improves contractility and Ca^2+^ cycling in cardiomyocytes. Isolated cardiomyocytes from 4‐month‐old wild‐type mice were cultured in normal glucose (NG) and high glucose (HG). (a) Representative traces of calcium flux (upper panel) and sarcomere length (lower panel) of cardiomyocytes cultured in low glucose, with or without the EP4 agonist CAY10598. Acute hyperglycemia resulted in significant decreases in (c) maximal velocity of shortening and (d) maximal velocity of relengthening. Additionally, acute hyperglycemia resulted in significant increase in (f) time to peak [Ca^2+^]_I_ and (g) Ca^2+^]_I_ decay. Acute treatment with the EP4 agonist CAY10598 resulted in increased (b) fractional shortening, (c) maximum velocity of shortening, and (d) maximum velocity of relengthening in normal (NG)‐ and high glucose (HG)‐cultured cardiomyocytes. Acute CAY10598 treatment also increased (e) [Ca^2+^]_i_ peak amplitude and accelerated (f) time to peak [Ca^2+^]_i_ and (G) [Ca^2+^]_i_ decay. Results are expressed as a fold of the steady‐state baseline of vehicle‐treated NG control (NG: Control). Data were analyzed using a two‐way ANOVA with Tukey multiple comparisons post hoc test. Statistics: Same colored symbols represent separate individual cardiomyocytes derived from the same heart. *N* = 15 cardiomyocytes from four hearts per group. ●: NG control, ⚪: NG DG‐041, ▴: HG control, ▵: HG DG‐041. x = vs NG control, * vs NG control, † vs HG control, $ vs NG DG041. 1 symbol: *p *< 0.05, 2 symbols: *p *< 0.01, 3 symbols: *p *< 0.001, 4 symbols: *p *< 0.0001

Because EP3 and EP4 exert opposing effects on cardiac function in vivo, we next sought to determine whether activation of the EP4 receptor caused similar effects on cardiomyocyte contractility and Ca^2+^ dynamics as blockade of EP3. Cardiomyocytes isolated from 4‐month‐old wild‐type mice were cultured in normal or high glucose conditions and electrically paced before being superfused with the EP4 agonist CAY10598 or vehicle control; representative traces are shown in Figure 3a. Fractional shortening was increased (1.23 +/− 0.03 vs 0.78 +/− 0.02; Figure 3b) and maximum velocity of shortening (1.33 +/− 0.05 vs 0.85 +/− 0.02; Figure 3c) and maximum velocity of relengthening (0.98 +/− 0.03 vs 0.70 +/− 0.02; Figure 3d) was accelerated in CAY10598‐treated cardiomyocytes cultured in high glucose. Cardiomyocytes cultured in high glucose and treated with the EP4 agonist displayed increased peak [Ca^2+^]_I_ amplitude (1.73 +/− 0.08 vs 0.91 +/− 0.05; Figure 3e), decreased time to peak [Ca^2+^]_I_ (0.97 +/− 0.04 vs 1.27 +/− 0.05; Figure 3f), and more rapid [Ca^2+^]_I_ decay (0.97 +/− 0.05 vs 1.37 +/− 0.03; Figure 3g). Similar improvements in contractile properties were observed in CAY10598‐treated cardiomyocytes cultured in normal glucose (Figure 3).

### 3.4 Ptger3 gene expression is decreased and Ptger4 gene expression is unchanged in hearts from db/db mice

**FIGURE 4:**
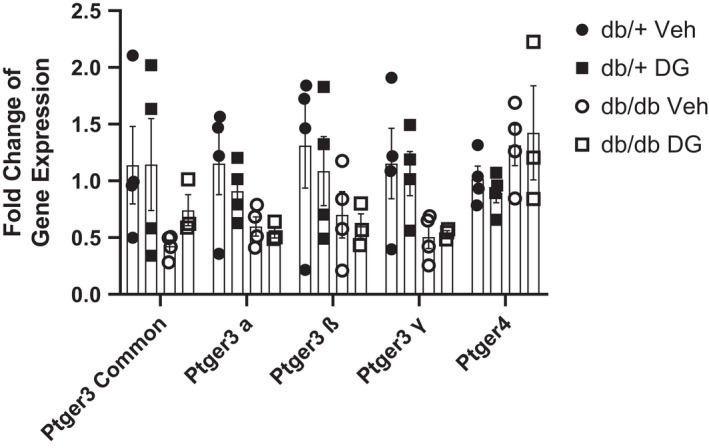
*Ptger3* and *Ptger4* expression in whole hearts of db/+ and db/db mice. Comparison of *Ptger3* and *Ptger4* expression in hearts isolated from 8‐week‐old db/+ and db/db after 2 weeks of vehicle (10% DMSO in PBS) or 20 mg/kg DG‐041 treatment. Gene expression was quantified relative to actin and then expressed relative to expression in vehicle‐treated db/+ mice. Results represent mean +/− S.E.M. *n* = 3–4 mice per group. ●, db/+ treated with vehicle. ◾, db/+ treated with DG‐041. ⚪, db/db treated with vehicle. ◽, db/db treated with DG‐041

The functional studies described above indicate that modulation of EP3 or EP4 activity can have beneficial effects on cardiomyocyte function. However, the expression levels of the genes encoding these receptors (*Ptger3* and *Ptger4*, respectively) in the heart, particularly within the context of T2D, have not been described. We performed qRT‐PCR on lysates from whole hearts isolated from 8‐week‐old db/+ and db/db mice. There was a trend toward decreased expression of total *Ptger3* in db/db mice, as well as a trend toward a decrease in the three known murine splice variants of *Ptger3* (Figure 4), whereas *Ptger4* expression was unchanged in db/db hearts compared with db/+ controls. We also assessed expression of these genes in 8‐week‐old db/+ and db/db mice that were treated daily with 20 mg/kg of DG‐041 in vivo for 14 days to determine if blockade of EP3 activity had any effect on the expression of *Ptger3* or *Ptger4*. In previous studies from our lab, we showed that this treatment paradigm has no effects on measurements of whole‐body glucose homeostasis, including glucose tolerance, insulin resistance, body weight, or fasting glucose levels (Bosma et al., 2021). DG‐041 treatment had no effect on the expression of total *Ptger3*, any *Ptger3* splice variant, or *Ptger4* in the heart (Figure 4). Given the lack of highly specific antibodies against EP3 and EP4 protein, we are unable to determine the expression levels of these receptors in cardiomyocytes.

### 3.5 In vivo blockade of EP3 causes modest improvements in cardiac function

**FIGURE 5:**
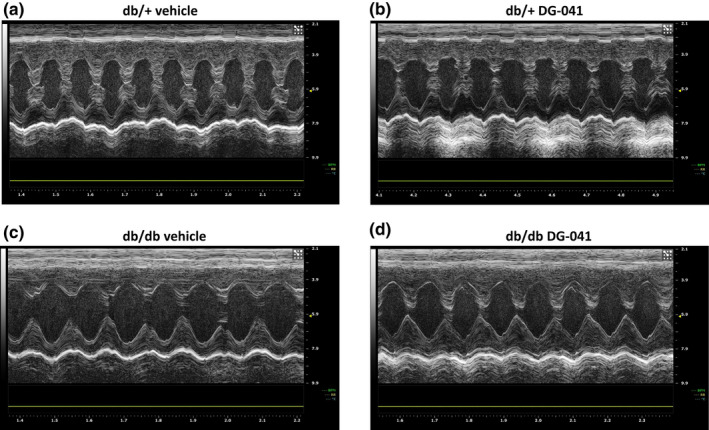
Echocardiography images from vehicle and DG‐041‐treated db/+ and db/db mice. Representative images from transthoracic echocardiography performed on 8‐week‐old (a) db/+ mice treated for 2 weeks with vehicle, (b) db/+ mice treated for 2 weeks with DG‐041, (c) db/db mice treated for 2 weeks with vehicle, and (d) db/db mice treated for 2 weeks with DG‐041. Images were obtained on conscious mice using a Vevo2100 Imaging System

**FIGURE 6:**
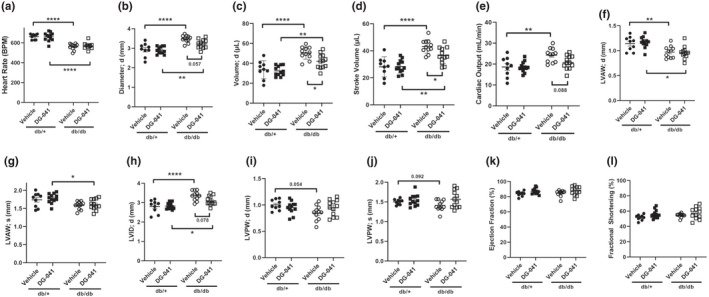
Cardiac function in vehicle and DG‐041‐treated db/+ and db/db mice. (a) Heart rate, (b) diastolic diameter, (c) diastolic volume, (d) stroke volume, (e) cardiac output, (f) LVAWd, (g) LVAWs, (h) LVIDd, (i) LVAWd, (j) LVAWs, (k) ejection fraction, and (l) fractional shortening in 8‐week‐old db/+ or db/db mice treated with either vehicle or DG‐041 for 2 weeks. Data points represent summarized data from three consecutive cardiac cycles. Summarized data were analyzed via ANOVA followed by Bonferroni post hoc analysis. Results represent mean +/− S.E.M. ●, db/+ treated with vehicle. ◾, db/+ treated with DG‐041. ⚪, db/db treated with vehicle. ◽, db/db treated with DG‐041

The results from our isolated cardiomyocyte studies indicated that EP3 antagonists or EP4 agonists elicit positive inotropic effects under normal and acute high glucose conditions. We next analyzed whether systemic treatment with EP3 antagonist in vivo would result in improved cardiac function in euglycemic and hyperglycemic mouse models. Six‐week‐old db/+ and db/db mice were treated subcutaneously with DG‐041 or vehicle control daily for 2 weeks and were then subject to transthoracic echocardiography. Representative images are shown in Figure 5. Heart rate (Figure 6a), diastolic diameter (Figure 6b), diastolic volume (Figure 6c), stroke volume (Figure 6d), cardiac output (Figure 6e), diastolic left ventricular anterior wall thickness (LVAWd, Figure 6f), and diastolic left ventricular interior diameter (LVIDd, Figure 6h) were all significantly different between db/db mice and the db/+ controls, indicative of impairment of cardiac function in the diabetic mice. Both diastolic and systolic left ventricular posterior wall thickness (LVPWd and LVPWs, Figure 6i,j) showed trends toward decreases in the vehicle‐treated db/db mice relative to their db/+ counterparts.

EP3 blockade resulted in significantly decreased left ventricular anterior wall thickness (LVAWs, Figure 6g) between genotypes but this effect was not observed between genotypes in the vehicle‐treated groups. There were no differences between genotypes in ejection fraction, fractional shortening, or LV mass (Figure 6k,l and data not shown). EP3 blockade had no effect in db/+ mice on any of the measured parameters, but db/db mice treated with DG‐041 showed modest improvements in diastolic diameter (Figure 6b), diastolic volume (Figure 6c), stroke volume (Figure 6d), cardiac output (Figure 6e), and LVIDd (Figure 6h). Taken together, these results indicate that short‐term blockade of EP3 in vivo has positive effects on cardiac function in diabetic mice.

## 4 DISCUSSION

T2D is associated with chronic low‐grade inflammation and elevated circulating prostaglandin levels, and is also associated with a two‐ to fourfold increased incidence of heart attack and cardiac death (Fox, 2010). COX‐2, and thus, PGE_2_ synthesis, is upregulated in human cardiomyocytes in the setting of inflammation, ischemia, and myocardial infarction (Bolli, 2002; Zidar et al., 2007). This elevation in PGE_2_ has been shown to have cardioprotective roles (Bolli et al., 2002; Wang et al., 2009). Thus, general COX inhibition by NSAIDs to reduce the chronic inflammation present in T2D can have deleterious consequences on cardiac function (Bleumink, Feenstra, Sturkenboom & Stricker, 2003; Marsico et al.,; Pepine & Gurbel, 2017). This is likely due to the loss of action of PGE_2_ through EP4 receptors, which mediate the beneficial effects of PGE_2_ on cardiac function. Since PGE_2_ binds both EP3 and EP4 with equal affinity (Sugimoto & Narumiya, 2007), the ultimate effects of PGE_2_ will depend on the expression levels of these receptors. We and others have found that T2D alters the balance of expression of EP3 and EP4 in pancreatic islets, with an increase in expression of EP3 (*Ptger3*) (Carboneau et al., 2017; Kimple et al., 2013). Therefore, identification of therapeutically relevant receptor‐specific agonists and antagonists is critical to achieve the desired physiological outcome of EP3 and EP4 modulation. Along those lines, we showed that specific blockade of EP3 in db/db mice in vivo enhanced proliferation of insulin‐producing β cells, increased β‐cell mass, and improved β‐cell gene expression (Bosma et al., 2021). Given the known opposing roles of EP3 and EP4 in cardiac function, therapies designed to selectively block EP3‐mediated PGE_2_ activity, while allowing for activation of EP4 might thus prove most beneficial.

In the current study, we investigated whether modulation of PGE_2_ receptor activity in the setting of hyperglycemia or overt T2D was beneficial to parameters of cardiomyocyte function known to be affected in diabetic cardiomyopathy. The db/db mouse model of T2D has been validated as a model of human diabetic cardiomyopathy, particularly heart failure with preserved ejection fraction (HFpEF), commonly seen in human obesity and diabetes. Db/db mice also have increased PGE_2_ levels, as observed in obesity in humans (Sun et al., 2013). We found that EP3 blockade or EP4 activation EP3 blockade in vivo had beneficial effects on cardiac function in db/db mice.

Although EP3 blockade improved fractional shortening in isolated cardiomyocytes, it did not improve fractional shortening in vivo in db/db mice. This could be due to the differences in the duration of treatment (5 min for the isolated cardiomyocytes versus 2 weeks of systemic treatment), or to additional effects of the hyperglycemic and hyperlipidemic environment in db/db mice. Additionally, since leptin plays a role in regulating cardiac function (Poetsch, Strano & Guan, 2020), the lack of improvements in ejection fraction and fractional shortening in db/db mice could be attributed to a loss of leptin signaling, although this possibility was not directly assessed here. It is also important to note that the observed effects of acute (5 min) EP3 antagonist treatment in isolated cardiomyocytes are likely due to alterations in second messenger signaling pathways rather than changes in gene or protein expression. EP3 receptor (*Ptger3*) expression was decreased in hearts from vehicle‐treated db/db mice compared to db/+ controls. However, since expression was assessed in extracts from whole hearts, the observed decrease in expression may be due to effects in cardiomyocytes and/or other cell types such as fibroblasts or endothelial cells. Further gene expression analyses in isolated cardiomyocytes will be needed to distinguish the specific effects of acute and chronic EP3 blockade on cardiomyocytes.

There was no effect of EP3 blockade on any of the cardiac function parameters we assessed in db/+ mice in vivo (which are euglycemic). Unexpectedly, EP3 blockade improved all cardiomyocyte functional parameters tested even when cultured under normal glucose conditions, suggesting that EP3 is tonically active in isolated cardiomyocytes under basal conditions, at least in culture. It is possible that, when placed in culture, cardiomyocytes release PGE_2_, which signals in an autocrine manner in this system.

Although db/db mice have been shown to have early evidence of cardiac dysfunction by 9 weeks of age, worsening heart function and increased collagen deposition, hallmarks of diabetic cardiomyopathy, occur between 12 and 16 weeks of age (Baumgardt et al., 2016; Nielsen et al., 2009). The current experiments were carried out in 8 week old mice after 2 weeks of treatment with the EP3 antagonist. This time point was chosen as it corresponded with a time point at which previous studies from our lab showed beneficial effects of in vivo EP3 blockade on β‐cell mass and identity (Bosma et al., 2021). In addition, this dose, duration, and route of administration result in plasma concentrations of EP3 antagonist that achieve complete blockade of the EP3 receptor in vivo (Ceddia et al., 2019). It is important to note that this treatment paradigm did not result in improvements in whole‐body glucose homeostasis or insulin resistance in db/db mice (Bosma et al., 2021), suggesting that the observed effects of EP modulators on cardiac function are direct rather than due to improvements in the systemic metabolic or physiologic milieu. While our data are promising, it is vital to examine if drug treatment would prove even more effective in older db/db mice where cardiac dysfunction is more apparent. We also plan to try combined in vivo treatment with EP3 antagonist and EP4 agonist as this strategy worked best to stimulate β‐cell proliferation in isolated human pancreatic islets (Carboneau et al., 2017).

The glucolipotoxicity associated with diabetes causes intrinsic defects in cardiomyocytes, including oxidative damage, that render them more susceptible to dysfunction in the setting of increased stress such as ischemia (Boudina & Abel, 2010; Brahma, Pepin & Wende, 2017; Selvin et al., 2014). We propose that altering the balance from EP3 to EP4 activity in the setting of diabetes will have significant beneficial functional consequences for the effects of PGE_2_ on cardiac tissue. GPCRs represent 35% of all current drug targets (Sriram & Insel, 2018), highlighting the potential of the EP receptors as future druggable targets for the treatment of T2D and its comorbidities. Our current model proposes that EP3 and EP4 play opposing roles in the heart and that shifting the balance toward increased EP4 activity will be beneficial in the setting of diabetic cardiomyopathy. The expression of EP3 and EP4 in human cardiomyocytes and their respective roles in diabetic cardiomyopathy have not yet been explored. EP receptors have great potential as future druggable targets for the treatment of T2D. Because the EP3 and EP4 receptors have been shown to have effects on β‐cell proliferation, survival, and function as well as cardiac function, we propose that modulating these receptors would have positive effects on both CVD and T2D, and thereby potentially treat both diseases simultaneously.

## AUTHOR CONTRIBUTIONS

S.R.A., M. Ghosh, D.S.D., and M. Gannon conceived the studies. K.J.B., S.R.A., M. Ghosh, L.Z., V.F.R., J.C.D., and J.B.B. performed experiments, analyzed the data, and generated figures. A.K.H., D.S.D., and M.G. also analyzed the data. S.R.A. wrote the first draft of the manuscript. K.J.B., M. Ghosh, A.K.H., D.S.D., and M.G. edited and finalized the manuscript.

## References

[phy215212-bib-0001] Abbasi, F. , Brown, B. W. , Lamendola, C. , McLaughlin, T. , & Reaven, G. M. (2002). Relationship between obesity, insulin resistance, and coronary heart disease risk. Journal of the American College of Cardiology, 40(5), 937–943. 10.1016/S0735-1097(02)02051-X 12225719

[phy215212-bib-0002] Alex, L. , Russo, I. , Holoborodko, V. , & Frangogiannis, N. G. (2018). Characterization of a mouse model of obesity‐related fibrotic cardiomyopathy that recapitulates features of human heart failure with preserved ejection fraction. American Journal of Physiology. Heart and Circulatory Physiology, 315(4), H934–H949. 10.1152/ajpheart.00238.2018 30004258 PMC6230908

[phy215212-bib-0003] Andrei, S. R. , Ghosh, M. , Sinharoy, P. , & Damron, D. S. (2019). Stimulation of TRPA1 attenuates ischemia‐induced cardiomyocyte cell death through an eNOS‐mediated mechanism. Channels (Austin), 13(1), 192–206. 10.1080/19336950.2019.1623591 31161862 PMC6557600

[phy215212-bib-0004] Andrei, S. R. , Ghosh, M. , Sinharoy, P. , Dey, S. , Bratz, I. N. , & Damron, D. S. (2017). TRPA1 ion channel stimulation enhances cardiomyocyte contractile function via a CaMKII‐dependent pathway. Channels (Austin), 11(6), 587–603. 10.1080/19336950.2017.1365206 28792844 PMC5786180

[phy215212-bib-0005] Baumgardt, S. L. , Paterson, M. , Leucker, T. M. , Fang, J. , Zhang, D. X. , Bosnjak, Z. J. , Warltier, D. C. , Kersten, J. R. , Ge, Z.‐D. (2016). Chronic co‐administration of sepiapterin and L‐citrulline ameliorates diabetic cardiomyopathy and myocardial ischemia/reperfusion injury in obese type 2 diabetic mice. Circulation: Heart Failure, 9(1), e002424. 26763290 10.1161/CIRCHEARTFAILURE.115.002424 PMC4714787

[phy215212-bib-0006] Belke, D. D. , Swanson, E. A. , & Dillmann, W. H. (2004). Decreased sarcoplasmic reticulum activity and contractility in diabetic db/db mouse heart. Diabetes, 53(12), 3201–3208. 15561951 10.2337/diabetes.53.12.3201

[phy215212-bib-0007] Bleumink, G. S. , Feenstra, J. , Sturkenboom, M. C. J. M. , & Stricker, B. H. C. (2003). Nonsteroidal anti‐inflammatory drugs and heart failure. Drugs, 63(6), 525–534. 10.2165/00003495-200363060-00001 12656651

[phy215212-bib-0008] Bolli, R. (2002). Discovery of a new function of cyclooxygenase (COX)‐2: COX‐2 is a cardioprotective protein that alleviates ischemia/reperfusion injury and mediates the late phase of preconditioning. Cardiovascular Research, 55(3), 506–519. 10.1016/S0008-6363(02)00414-5 12160947 PMC3242376

[phy215212-bib-0009] Bosma, K. J. , Andrei, S. R. , Katz, L. S. , Smith, A. A. , Dunn, J. C. , Ricciardi, V. F. , Ramirez, M. A. , Baumel‐Alterzon, S. , Pace, W. A. , Carroll, D. T. , Overway, E. M. , Wolf, E. M. , & Kimpl, M. E. (2021). Pharmacological blockade of the EP3 prostaglandin E2 receptor in the setting of type 2 diabetes enhances beta‐cell proliferation and identity and relieves oxidative damage. Molecular Metabolism, 54, 101347. 34626853 10.1016/j.molmet.2021.101347 PMC8529552

[phy215212-bib-0010] Boudina, S. , & Abel, E. D. (2010). Diabetic cardiomyopathy, causes and effects. Reviews in Endocrine & Metabolic Disorders, 11(1), 31–39. 10.1007/s11154-010-9131-7 20180026 PMC2914514

[phy215212-bib-0011] Brahma, M. K. , Pepin, M. E. , & Wende, A. R. (2017). My sweetheart is broken: Role of glucose in diabetic cardiomyopathy. Diabetes & Metabolism Journal, 41(1), 1–9. 10.4093/dmj.2017.41.1.1 28236380 PMC5328690

[phy215212-bib-0012] Bryson, T. D. , Gu, X. , Khalil, R. M. , Khan, S. , Zhu, L. , Xu, J. , Peterson, E. , Yang, X.‐P. , & Harding, P. (2018). Overexpression of prostaglandin E2 EP4 receptor improves cardiac function after myocardial infarction. Journal of Molecular and Cellular Cardiology, 118, 1–12. 10.1016/j.yjmcc.2018.03.005 29522761 PMC5940572

[phy215212-bib-0013] Bugger, H. , & Abel, E. D. (2014). Molecular mechanisms of diabetic cardiomyopathy. Diabetologia, 57(4), 660–671. 10.1007/s00125-014-3171-6 24477973 PMC3969857

[phy215212-bib-0014] Carboneau, B. A. , Allan, J. A. , Townsend, S. E. , Kimple, M. E. , Breyer, R. M. , & Gannon, M. (2017). Opposing effects of prostaglandin E2 receptors EP3 and EP4 on mouse and human beta‐cell survival and proliferation. Molecular Metabolism, 6(6), 548–559. 28580285 10.1016/j.molmet.2017.04.002 PMC5444094

[phy215212-bib-0015] Ceddia, R. P. , Downey, J. D. , Morrison, R. D. , Kraemer, M. P. , Davis, S. E. , Wu, J. , Lindsley, C. W. , Yin, H. , Daniels, J. S. , & Breyer, R. M. (2019). The effect of the Ep3 antagonist Dg‐041 on male mice with diet‐induced obesity. Prostaglandins & Other Lipid Mediators, 144, 106353. 10.1016/j.prostaglandins.2019.106353 31276827 PMC6778036

[phy215212-bib-0016] de Simone, G. , Devereux, R. B. , Chinali, M. , Lee, E. T. , Galloway, J. M. , Barac, A. , Panza, J. A. , & Howard, B. V. (2010). Diabetes and incident heart failure in hypertensive and normotensive participants of the Strong Heart Study. Journal of Hypertension, 28(2), 353–360. 10.1097/HJH.0b013e3283331169 19844184 PMC3005764

[phy215212-bib-0017] Devereux, R. B. , Roman, M. J. , Liu, J. E. , Welty, T. K. , Lee, E. T. , Rodeheffer, R. , Fabsitz, R. R. , Howard, B. V. (2000). Congestive heart failure despite normal left ventricular systolic function in a population‐based sample: The Strong Heart Study. American Journal of Cardiology, 86(10), 1090–1096. 10.1016/s0002-9149(00)01165-6 11074205

[phy215212-bib-0018] Dyntar, D. , Sergeev, P. , Klisic, J. , Ambühl, P. , Schaub, M. C. , & Donath, M. Y. (2006). High glucose alters cardiomyocyte contacts and inhibits myofibrillar formation. Journal of Clinical Endocrinology and Metabolism, 91(5), 1961–1967. 10.1210/jc.2005-1904 16522700

[phy215212-bib-0019] Fox, C. S. (2010). Cardiovascular disease risk factors, type 2 diabetes mellitus, and the Framingham Heart Study. Trends in Cardiovascular Medicine, 20(3), 90–95. 10.1016/j.tcm.2010.08.001 21130952 PMC3033760

[phy215212-bib-0020] Gu, X. , Xu, J. , Zhu, L. , Bryson, T. , Yang, X.‐P. , Peterson, E. , & Harding, P. (2016). Prostaglandin E2 reduces cardiac contractility via EP3 receptor. Circulation: Heart Failure, 9(8). 10.1161/CIRCHEARTFAILURE.116.003291 PMC4979610 27502370

[phy215212-bib-0021] Guellich, A. , Mehel, H. , & Fischmeister, R. (2014). Cyclic AMP synthesis and hydrolysis in the normal and failing heart. Pflugers Archiv—european Journal of Physiology, 466(6), 1163–1175. 10.1007/s00424-014-1515-1 24756197

[phy215212-bib-0022] Hata, A. N. , & Breyer, R. M. (2004). Pharmacology and signaling of prostaglandin receptors: Multiple roles in inflammation and immune modulation. Pharmacology & Therapeutics, 103(2), 147–166. 10.1016/j.pharmthera.2004.06.003 15369681

[phy215212-bib-0023] Hishikari, K. , Suzuki, J.‐I. , Ogawa, M. , Isobe, K. , Takahashi, T. , Onishi, M. , Takayama, K. , & Isobe, M. (2009). Pharmacological activation of the prostaglandin E2 receptor EP4 improves cardiac function after myocardial ischaemia/reperfusion injury. Cardiovascular Research, 81(1), 123–132. 10.1093/cvr/cvn254 18805784 PMC2721641

[phy215212-bib-0024] Jia, G. , Hill, M. A. , & Sowers, J. R. (2018). Diabetic cardiomyopathy: An update of mechanisms contributing to this clinical entity. Circulation Research, 122(4), 624–638. 10.1161/CIRCRESAHA.117.311586 29449364 PMC5819359

[phy215212-bib-0025] Kimple, M. E. , Keller, M. P. , Rabaglia, M. R. , Pasker, R. L. , Neuman, J. C. , Truchan, N. A. , Brar, H. K. , & Attie, A. D. (2013). Prostaglandin E2 receptor, EP3, is induced in diabetic islets and negatively regulates glucose‐ and hormone‐stimulated insulin secretion. Diabetes, 62(6), 1904–1912. 10.2337/db12-0769 23349487 PMC3661627

[phy215212-bib-0026] Kralik, P. M. , Ye, G. , Metreveli, N. S. , Shen, X. , & Epstein, P. N. (2005). Cardiomyocyte dysfunction in models of type 1 and type 2 diabetes. Cardiovascular Toxicology, 5(3), 285–292. 10.1385/CT:5:3:285 16244373

[phy215212-bib-0027] Lang, R. M. , Bierig, M. , Devereux, R. B. , Flachskampf, F. A. , Foster, E. , Pellikka, P. A. , Picard, M. H. , Roman, M. J. , Seward, J. , Shanewise, J. S. , Solomon, S. D. , Spencer, K. T. , St John Sutton, M. , & Stewart, W. J. (2005). Recommendations for chamber quantification: a report from the American Society of Echocardiography's Guidelines and Standards Committee and the Chamber Quantification Writing Group, developed in conjunction with the European Association of Echocardiography, a branch of the European Society of Cardiology. Journal of the American Society of Echocardiography, 18(12), 1440–1463. 10.1016/j.echo.2005.10.005 16376782

[phy215212-bib-0028] Marsico, F. , Paolillo, S. , & Filardi, P. P. (2017) NSAIDs and cardiovascular risk. Journal of Cardiovascular Medicine, 18(Suppl. 1): Special Issue on The State of the Art for the Practicing Cardiologist: The 2016 Conoscere E Curare Il Cuore (CCC), Proceedings from the CLI Foundation. pp. e40–e43. 10.2459/JCM.0000000000000443 27652819

[phy215212-bib-0029] Meyer‐Kirchrath, J. , Martin, M. , Schooss, C. , Jacoby, C. , Flogel, U. , Marzoll, A. , Fischer, J. W. , Schrader, J. , Schror, K. , & Hohlfeld, T. (2009). Overexpression of prostaglandin EP3 receptors activates calcineurin and promotes hypertrophy in the murine heart. Cardiovascular Research, 81(2), 310–318. 10.1093/cvr/cvn312 19019835

[phy215212-bib-0030] Nielsen, J. M. , Kristiansen, S. B. , Nørregaard, R. , Andersen, C. L. , Denner, L. , Nielsen, T. T. , Flyvbjerg, A. , & Bøtker, H. E. (2009). Blockage of receptor for advanced glycation end products prevents development of cardiac dysfunction in db/db type 2 diabetic mice. European Journal of Heart Failure, 11(7), 638–647. 10.1093/eurjhf/hfp070 19502378

[phy215212-bib-0031] Pepine, C. J. , & Gurbel, P. A. (2017). Cardiovascular safety of NSAIDs: Additional insights after PRECISION and point of view. Clinical Cardiology, 40(12), 1352–1356. 10.1002/clc.22814 29247518 PMC6490377

[phy215212-bib-0032] Plante, E. , Menaouar, A. , Danalache, B. A. , Broderick, T. L. , Jankowski, M. , & Gutkowska, J. (2014). Treatment with brain natriuretic peptide prevents the development of cardiac dysfunction in obese diabetic db/db mice. Diabetologia, 57(6), 1257–1267. 10.1007/s00125-014-3201-4 24595856

[phy215212-bib-0033] Plante, E. , Menaouar, A. , Danalache, B. A. , Yip, D. , Broderick, T. L. , Chiasson, J.‐L. , Jankowski, M. , & Gutkowska, J. (2015). Oxytocin treatment prevents the cardiomyopathy observed in obese diabetic male db/db mice. Endocrinology, 156(4), 1416–1428. 10.1210/en.2014-1718 25562615

[phy215212-bib-0034] Poetsch, M. S. , Strano, A. , & Guan, K. (2020). Role of leptin in cardiovascular diseases. Frontiers in Endocrinology (Lausanne), 11, 354. 10.3389/fendo.2020.00354 PMC7325922 32655492

[phy215212-bib-0035] Qian, J. Y. , Harding, P. , Liu, Y. , Shesely, E. , Yang, X.‐P. , & LaPointe, M. C. (2008). Reduced cardiac remodeling and function in cardiac‐specific EP4 receptor knockout mice with myocardial infarction. Hypertension, 51(2), 560–566. 18180401 10.1161/HYPERTENSIONAHA.107.102590 PMC3115709

[phy215212-bib-0036] Selvin, E. , Lazo, M. , Chen, Y. , Shen, L. , Rubin, J. , McEvoy, J. W. , Hoogeveen, R. C. , Sharrett, A. R. , Ballantyne, C. M. , & Coresh, J. (2014). Diabetes mellitus, prediabetes, and incidence of subclinical myocardial damage. Circulation, 130(16), 1374–1382. 10.1161/CIRCULATIONAHA.114.010815 25149362 PMC4198442

[phy215212-bib-0037] Sjoholm, A. , & Nystrom, T. (2006). Inflammation and the etiology of type 2 diabetes. Diabetes/Metabolism Research and Reviews, 22(1), 4–10. 10.1002/dmrr.568 15991254

[phy215212-bib-0038] Sriram, K. , & Insel, P. A. (2018). G protein‐coupled receptors as targets for approved drugs: How many targets and how many drugs? Molecular Pharmacology, 93(4), 251–258. 29298813 10.1124/mol.117.111062 PMC5820538

[phy215212-bib-0039] Sugimoto, Y. , & Narumiya, S. (2007). Prostaglandin E receptors. Journal of Biological Chemistry, 282(16), 11613–11617. 10.1074/jbc.R600038200 17329241

[phy215212-bib-0040] Sun, Y. , Jia, Z. , Liu, G. , Zhou, L. , Liu, M. , Yang, B. , & Yang, T. (2013). PPARγ agonist rosiglitazone suppresses renal mPGES‐1/PGE2 pathway in db/db mice. PPAR Research, 2013, 612971. 24489534 10.1155/2013/612971 PMC3892750

[phy215212-bib-0041] Vennemann, A. , Gerstner, A. , Kern, N. , Ferreiros Bouzas, N. , Narumiya, S. , Maruyama, T. , & Nüsing, R. M. (2012). PTGS‐2‐PTGER2/4 signaling pathway partially protects from diabetogenic toxicity of streptozotocin in mice. Diabetes, 61(7), 1879–1887. 10.2337/db11-1396 22522619 PMC3379658

[phy215212-bib-0042] Wang, D. , Patel, V. V. , Ricciotti, E. , Zhou, R. , Levin, M. D. , Gao, E. , Yu, Z. , Ferrari, V. A. , Lu, M. M. , Xu, J. , Zhang, H. , Hui, Y. , Cheng, Y. , Petrenko, N. , Yu, Y. , & FitzGerald, G. A. (2009). Cardiomyocyte cyclooxygenase‐2 influences cardiac rhythm and function. Proceedings of the National Academy of Sciences of the United State of America, 106(18), 7548–7552. 10.1073/pnas.0805806106 PMC2670242 19376970

[phy215212-bib-0043] Wickley, P. J. , Shiga, T. , Murray, P. A. , & Damron, D. S. (2007). Propofol modulates Na+‐Ca2+ exchange activity via activation of protein kinase C in diabetic cardiomyocytes. Anesthesiology, 106(2), 302–311. 17264725 10.1097/00000542-200702000-00019

[phy215212-bib-0044] Zidar, N. , Dolenc‐Stražar, Z. , Jeruc, J. , Jerše, M. , Balažic, J. , Gartner, U. , Jermol, U. , Zupanc, T. , & Štajer, D. (2007). Expression of cyclooxygenase‐1 and cyclooxygenase‐2 in the normal human heart and in myocardial infarction. Cardiovasc Pathol, 16(5), 300–304. 10.1016/j.carpath.2007.02.005 17868881

